# A Smart City Lighting Case Study on an OpenStack-Powered Infrastructure

**DOI:** 10.3390/s150716314

**Published:** 2015-07-06

**Authors:** Giovanni Merlino, Dario Bruneo, Salvatore Distefano, Francesco Longo, Antonio Puliafito, Adnan Al-Anbuky

**Affiliations:** 1 Mobile and Distributed Systems Lab, Dipartimento di Ingegneria, Universita di Messina, Contrada di Dio, 98166 Messina, Italy; E-Mails: dbruneo@unime.it (D.B.); flongo@unime.it (F.L.); apuliafito@unime.it (A.P.); 2 Dipartimento DIEEI, Universita di Catania, Viale Andrea Doria 6, 98166 Catania, Italy; 3 Social and Urban Computing Group, Higher Institute of Information Technologies and Information Systems, Kazan Federal University, 35 Kremlevskaya street, 420008 Kazan, Russia; E-Mail: s_distefano@it.kfu.ru; 4 Dipartimento DEIB, Politecnico di Milano, Piazza L. Da Vinci 32, 20133 Milano, Italy; 5 Sensor Network & Smart Environment Research Lab, School of Engineering, Auckland University of Technology, 34 St Paul Street (City Campus), Auckland 1010, New Zealand; E-Mail: adnan.anbuky@aut.ac.nz

**Keywords:** IoT, smart city, cloud, IaaS, OpenStack, ceilometer, MOM, AMQP, CoAP, REST, CEP

## Abstract

The adoption of embedded systems, mobile devices and other smart devices keeps rising globally, and the scope of their involvement broadens, for instance, in smart city-like scenarios. In light of this, a pressing need emerges to tame such complexity and reuse as much tooling as possible without resorting to vertical *ad hoc* solutions, while at the same time taking into account valid options with regard to infrastructure management and other more advanced functionalities. Existing solutions mainly focus on core mechanisms and do not allow one to scale by leveraging infrastructure or adapt to a variety of scenarios, especially if actuators are involved in the loop. A new, more flexible, cloud-based approach, able to provide device-focused workflows, is required. In this sense, a widely-used and competitive framework for infrastructure as a service, such as OpenStack, with its breadth in terms of feature coverage and expanded scope, looks to fit the bill, replacing current application-specific approaches with an innovative application-agnostic one. This work thus describes the rationale, efforts and results so far achieved for an integration of IoT paradigms and resource ecosystems with such a kind of cloud-oriented device-centric environment, by focusing on a smart city scenario, namely a park smart lighting example, and featuring data collection, data visualization, event detection and coordinated reaction, as example use cases of such integration.

## 1. Introduction

In recent years, different approaches, also based on new technologies, have been adopted to morph cities into smart ones. The smart city scenario is a fertile application domain for different sciences and technologies, in particular for those related to the information and communication areas. Several ongoing projects illustrate smart city opportunities and challenges [1] in diverse application fields, e.g., networking, decision support-systems, power grids, energy-aware platforms, service-oriented architectures, highlighting the need to equip the cities of the future with a variety of urban sensors. Plenty of applications can be envisioned, from traffic monitoring to energy management, from e-health to e-government, from crowd to emergency management. This all-encompassing and very ambitious scenario calls for adequate ICT technologies. In particular, solutions for managing the underlying physical sensing and actuation resources infrastructure are required.

A number of solutions of this kind, mainly at a lower (communication) layer, may be found within the Internet of Things (IoT) [2] domain, mainly aiming at interconnecting network-enabled devices and, generally, any thing featuring a network interface to the Internet. However, to manage devices, sensors and things building up a dynamic smart city infrastructure, organization and coordination mechanisms are also required. So far, related approaches mainly focus on the high application level, providing mechanisms and functionalities to collect, manage and process sensed data, which can be categorized as data centric. Anyway, to unleash IoT's potential, allowing things and/or service users to effectively interact each other and/or with the (surrounding) environment, as well, the data-centric paradigm is not enough; control capabilities on the devices should be provided to applications and services, which should be able to customize the underlying sensing infrastructure. This is particularly true when the interaction is triggered by the things or users (client-side), thus implying and involving actuators in the game. The new scenario thus established enables a degree of freedom in context-aware, augmented reality, crowd-based and, in general, any IoT-related application domain and could be framed in a device-centric approach. According to this novel perspective, an application or service has control of the sensing device, not only on the data that it provides.

To implement a device-centric approach for IoT and smart cities, cloud computing facilities, adopting a service-oriented approach in the provisioning and management of resources, may be exploited as a stepping stone to the generalization of otherwise custom approaches to the design of smart city applications. Indeed, the cloud could be a good solution to address smart city-related issues, fitting with the requirements of relevant service users and providers. Such requirements may include on-demand, elastic and QoS-guaranteed provisioning, as well as resource management, configuration and orchestration, to name a few, all needed properties for a smart city service platform.

To fill this gap between smart city applications and the underlying infrastructure, in this paper, we propose to adopt the device-centric approach, by extending a well-known framework for the management of cloud computing resources, OpenStack, to manage sensing and actuation ones. This way, we implement in the Stack4Things solution, an infrastructure-oriented [3] paradigm, coping with communication requirements and scalability concerns by leveraging cloud-focused design choices and architectural patterns. We demonstrate the effectiveness of the proposed framework by showing how it can be applied to a real-world scenario, namely a park smart lighting application.

For this purpose, the remainder of the paper is organized as follows. Section 2 reports the state-of-the-art in the context of smart cities and cloud integration, with specific regard to smart lighting applications and case studies. Section 3 formally describes the smart city scenario and investigates it from an infrastructure-focused perspective, also presenting our running scenario. In Section 4, we report the enabling technologies exploited, and the Stack4Things architecture is presented there with technical details and design decisions of the framework components implemented so far. Section 5 reports how the Stack4Things framework has been exploited in the implementation of our real-world running scenario. Finally, Section 6 concludes the paper with remarks on possible future work.

## 2. Related Work

Several works deal with infrastructure issues and solutions related to smart cities and their relationship with IoT and the cloud, both from open academic and commercial contexts. As an example, in [4], a platform for managing urban services that include convenience, health, safety and comfort is proposed. Furthermore, the cloud computing infrastructure recently [5,6] found useful application in the context of smart cities, but mainly as a complementary technology to support the processing and storage of sensed data. Similarly, the convergence of cloud and IoT and, in particular, the solutions to scale up IoT applications and to support real-time analytics have been thoroughly investigated during the last few years. A significant attempt in this direction is fog computing [7], where both IoT and cloud computing technologies are merged to provide new location-aware, reduced latency and improved QoS pervasive and ubiquitous services. Furthermore, based on this idea, several academic prototypes [8–11] and commercial offerings, such as xively [12], ThingWorx [13] or SmartThings Open Cloud [14], are already available.

As discussed above, all of these efforts can be therefore categorized in the data-centric paradigm, mainly aiming at managing (IoT sensed) data by the cloud. Even if many applications in the smart city scenario have been proposed so far, there is a lack of common initiatives and strategies to address issues at an infrastructural level also dealing with actuators, but as highlighted above, a data-centric perspective at a high application level is mainly taken into account. In [3], a different trajectory to tackle transducers in a cloud environment is taken: sensing and actuation devices should be handled along the same lines as computing and storage abstractions in traditional clouds, *i.e.*, virtualized and multiplexed over (scarce) hardware resources. In other words, instead of considering the cloud as a complementary technology, the proposed approach focuses on the service-oriented/cloud paradigm, adopting it in the management and provision of sensing resources and things, in a device-centric perspective. This step paves the way to an IoT/cloud-powered smart city framework able to support related services in the (sensing) infrastructure provisioning. A core highlight here lies in the design of sensing activities modelled as “probes” and the detection/reaction logic (to drive actuators) shifted to cloud-hosted event processors, which coupled together lead to both a higher degree of independence of the solution from the specific application domain and the treatment of generated data as metadata of cloud instances, the latter of which may ultimately aid in the actualization of an IaaS-oriented sensing/actuating cloud strategy.

In the specific context of smart lighting, an interesting work is [15], where a case study on a lighting system on an area not covered by the Internet has been fully developed by merging several lamps, sensors, power supply technologies and infrastructures through an intelligent management. The effectiveness of the solution in terms of both costs and reliability has been demonstrated. In a broader context, the relevance of the smart lighting control to reach the sustainability of smart cities is highlighted in [16], proposing an interoperable smart lighting solution based on Machine-to-Machine (M2M) protocols, such as CoAP over REST. From an abstract communication theoretic perspective, a hierarchical wireless network architecture of a smart lighting systems is proposed in [17], starting from IoT approaches and solutions, as well as on sensors and mobile technologies. A survey of networking solutions for smart lighting is provided in [18], including up-to-date sensing and networking technologies.

All of the above referred works deal with the problem of city lighting mainly defining *ad hoc*, specific, more or less smart solutions. In this paper, we propose to tackle the problem from a different, high level, broader perspective, *i.e.*, by adopting an infrastructural approach to establish a smart city infrastructure interconnecting all of the city devices and to provide it to smart lighting applications in a service-oriented, cloud-based fashion. This way, we have laid out in [19] the details of the first step in the direction of standardized, cross-domain approaches, where the focus was on integrating an open source framework for cloud management, OpenStack, with the IoT, in particular addressing the data collection and visualization stages by leveraging existing functionalities and the built-in scalability of the framework.

## 3. An IT Infrastructure for Smart Cities

**Figure 1:**
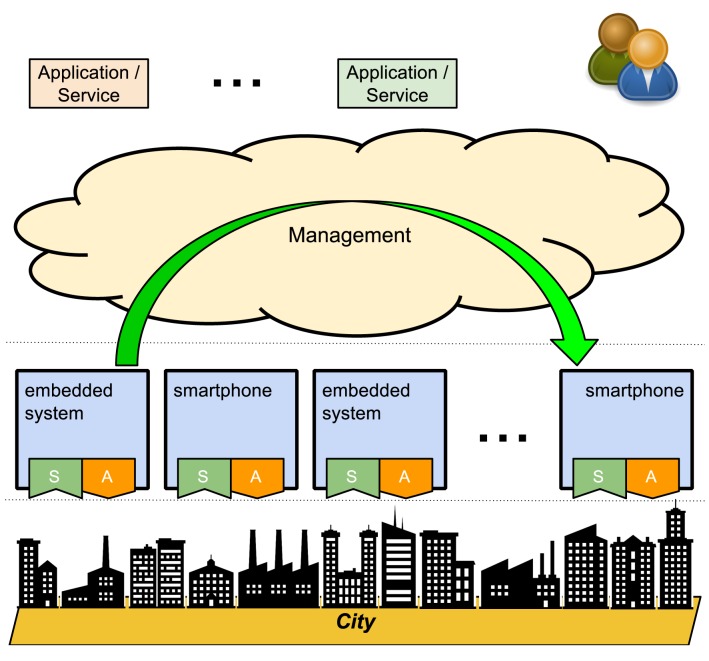
Smart city as a closed-loop system.

The smart city scenario is one of the most prominent scenarios for IoT involving computer-assisted treatment of sensed data and automation of urban areas and public facilities. Smart cities are usually characterized by field-deployed, dispersed, sensor-hosting smart platforms, usually available in several orders of magnitude greater quantities, possibly heterogeneous along several axes (instruction set architecture, operating system, user space, runtime, *etc.*). What makes a city smart is, apart from green and environment-friendly technologies, at least sensing and communication facilities, also embracing mobility, to enhance public transportation systems and monitor physical infrastructure (buildings, open spaces, *etc.*). When actuating subsystems enter the picture, the city as a whole may be compared to a nervous system, where reactive mechanisms are in place, either mediated centrally or even working autonomously. Indeed, as shown in Figure 1, a middleware devoted to management of both sensor- and actuator-hosting resources may help in the establishment of higher level services, including policies for “closing the loop”, such as configuring triggers for a range of (dispersed) actuators based on sensing activities from (geographically non-overlapping) sensing resources.

The data-centric solutions discussed in the previous section are not effective in this case, since they are not able to deal with the actuation, *i.e.*, they are just one way (device-to-application), whereas to close the loop, we need a bidirectional paradigm, able to also cope with application-to-device interactions. The only way to do that is by approaching the problem at a low, infrastructure level, adopting a device-centric approach for the sensing resource management, for example through a sensing cloud, as discussed in the following.

### 3.1. Exploiting the Cloud in a Smart City Context

At the other end of the scale, we have this recurring paradigm of typically outsourced, centralized services over which to delegate most computation and storage needs, going under the umbrella term of cloud computing [20]. We believe that, yet for all of the distance between the IoT and cloud perspectives, a wide range of synergies and opportunities lies at the intersection of the cloud and IoT, especially in the contest of smart cities. Indeed, what is needed for a smart city platform is at least a (virtual) infrastructure manager (VIM) over which to overlay higher level services, starting from the most basic ones, such as data collection, in order to extract value or possibly monetize the investments. In fact, in terms of requirements, it follows that any IT infrastructure powering smart cities features IoT-related devices, as well as typical data centres.

In line with recent trends and the latest advances, this means, on the one hand, resorting to private (or hybrid) cloud-enabled infrastructure, if not totally outsourcing computing and storage requirements to any of the established public IaaS providers. On the other hand, embedded systems are getting ever more powerful and flexible in terms of reprogrammable behaviour and ease of use. Indeed, most devices of such a kind are gaining a “smart” labelling to indicate this evolution.

A compelling example of the power a cloud middleware and the corresponding infrastructure may lend to such a scenario is the leverage cloud-enabled messaging and metering collection facilities provide for the design of data collection and visualization mechanisms, as exemplified later on in this paper. We foresee a fully infrastructure-oriented involvement of sensor/actuator-hosting boards further along this trajectory, as (virtual) objects to be mapped as managed resources in a pool.

Aiding the authors in framing the requirements for such a scenario, there is the early involvement in the *#SmartME* project [21], dealing with the enabling of the municipality of Messina as a smart city. Under the umbrella term of *#SmartME* lie both an effort to fund and procure hardware (thousands of boards and relative transducers) from a range of channels, including crowd funding, and a plan to deploy and set up such a huge IoT dust over a district-wide urban area.

In particular, *#SmartME* is meant both as an initiative to build an experimental test bed for smart city-relevant research and development and as the first iteration of joint research efforts over a wide range of use cases, one of which has been preliminarily investigated in this work. Other interesting use cases under investigation are: public transportation systems, environmental monitoring and mapping, personal mobility applications, and so on.

As a very high-level description of the requirements, the ability to identify, spot (on a map) and check devices for availability and overall health status, as well as to retrieve measurements on a variety of real-world phenomena, to be stored and visualized either for real-time consumption or long-term analysis for trends and other statistics to be inferred, is to be provided for a working smart city implementation. Measurements should also be analysed in real time to detect specific user-defined situations and trigger reactions in the form of actuation actions to be started and controlled by the central management system. In this context, scalability is also an essential requirement, at least after early experimentation and pilot testing, when getting into production stages.

Taking into account other experiences, such as the earliest experiments around smart city planning, e.g., projects like SmartSantander [22], typically, most efforts revolve around managing heterogeneous devices, usually by resorting to legacy protocols and vertical solutions out of necessity and integrating the whole ecosystem by means of an *ad hoc* solution. In our vision, the cloud may play a role both as a paradigm and as one or more ready-made solutions for a VIM, to be extended for IoT infrastructure. This way, on the one hand, the device-centric cloud solution addresses the heterogeneity issues though abstraction, and on the other hand, it provides a more flexible and elastic way to manage the sensing resources according to both the application and the provider requirements, through virtualization, placement and orchestration facilities, as detailed in the next section.

Our design is rooted in the notion that smart city infrastructure and the supporting cloud(s) are to be employed in a generalized way in order not to redesign the whole architecture when switching to different application domains and to support the concurrent exploitation of shared sensing/actuation facilities for multiple urban-related functions. An example of this application-agnostic approach may lie in the usage of CEPs in lieu of custom logic, in order to implement a range of applications in terms of statements to be hosted on the cloud and supported by the dispersed infrastructure.

### 3.2. Running Example: Park Smart Lighting

**Figure 2:**
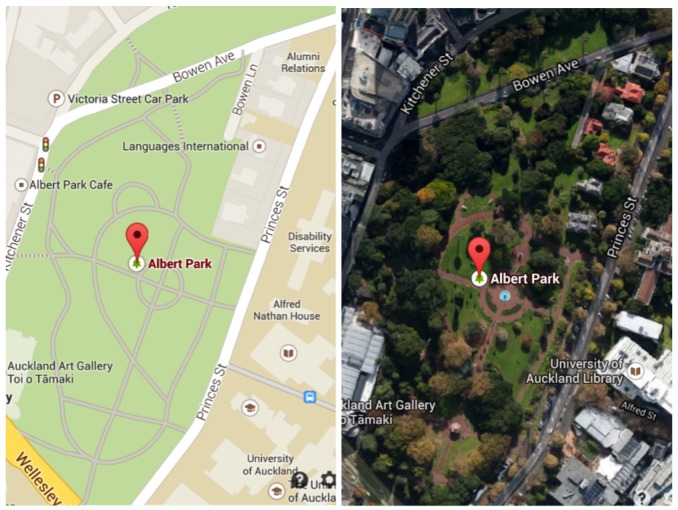
Google Maps and Google Earth screenshots of Albert Park in Auckland, New Zealand.

Monitoring of shared spaces, like a public green park, a zoo or a shopping mall, may provide a significant number of ancillary services. These include, but are not limited to, collecting information on functional performance, public security and safety-related data, infrastructure and maintenance requirements, public interest in remotely exploring the space, just to name a few. Lampposts in outdoor shared spaces may be considered as suitable receptacles belonging to the public areas that could host the infrastructure for certain monitoring systems. These are usually available in all kinds of public shared spaces in modern cities. In addition to access to lighting and related energy management, these may also provide access to energy sources and, thus, possibly leverage power line-based communication over the smart grid. If we take Albert Park as an example, at the heart of Auckland City in New Zealand (see Figure 2), we have an instance of a park serving, apart from residents from neighbouring areas, a big community belonging to two universities (more than 50,000 people). These are adjacent to the park, from both the east and south sides. The park is well organized, populated with plants, pedestrian and bicycle tracks, lampposts and other facilities. It provides a short cut for the people of the two universities to reach the city centre and main transport stations, like bus, train and ferry terminals.

Occasionally, the park presents some challenges in terms of keeping the area safe at night. This has called, over time, for the presence of security personnel randomly after sunset, leading to non-negligible costs for the tax payer. The availability of monitoring systems that can provide early warning on any significant event may help remarkably in keeping security inside the park at an acceptable level. Conventional approaches to the use of lampposts consist mostly of static lighting configurations.

**Figure 3:**
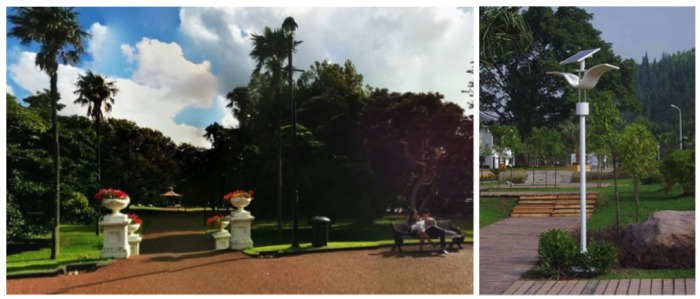
Standard lamp used currently at the park **(Left);** possible replacement with a modern LED light **(Right).**

Recent work encourages enhancements to the infrastructure, through sensing, actuation and communication, to provide other services [23]. Networking and communication facilities have been considered from a maintenance and operational perspective [24,25]. A more comprehensive approach to the exploitation of facilities calls for the integration of sensing, actuation and networking for interaction with spaces and users. A number of objectives may be targeted here, including energy saving and shared space safety [23]. Here, each lamppost could be equipped with, or connected to, heterogeneous sensors, like sound, light, human presence, and image sensors, in addition to LED lamps (see Figure 3). Such heterogeneous sensing provides some form of cooperative behaviour through the activation of a subset of the sensors and/or actuators, according to information originating from another subset of sensors. For example, a human presence sensor coupled with the detection of high sound intensity may indicate that there is an event that should be captured by the camera.

Another example is that the presence of an event during darkness calls for the activation of some artificial light sources. Energy harvesting devices, like a solar panel-based power supply, may supplement part or all of the required energy. Such a complex system may also include some form of data storage, processing and data communication for preliminary local data processing and absorbing any latency in data communication. A lamppost may also be considered as a single point of connectivity to the Internet and be part of the IoT. Here, remote users, like security personnel, could exploit the capability of the devices in capturing more layered information about the scene. This in effect helps with reducing the collection of sheer data to when and where it is needed.

An essential requirement lies then, on the one hand, in helping security and enforcement officials to be alerted when a situation of danger is occurring and, on the other, in letting the lighting system react on its own, which is where our IaaS-oriented approach fits in, by recycling frameworks and facilities for communication and centralized management, including remote access and visualization, while at the same time hosting complex event processing capabilities, the latter serving both visualization duties and autonomous mechanisms.

## 4. Stack4Things: Design and Implementation

In this section, we are going to describe our implementation efforts, geared toward data collection, data visualization, event detection and coordinated reaction as foremost examples of the kind of requirements to be met to provide a workable smart city-enabling solution.

### 4.1. Enabling Technologies

In the pursuit for integration of Cloud technologies with IoT infrastructure, we are trying to follow a bottom-up approach, consisting of a mixture of relevant, working frameworks and protocols, on one hand, and interesting use cases to be explored according to such integration effort.

Indeed, beyond concerns about the scale of the effort, other requirements such as elasticity of the sensing-based services to be provided, as well as registration and provisioning mechanisms of the underlying heterogeneous sensor-hosting platforms deserve an Infrastructure Manager (IM) anyway. For instance, scalability alone calls for a communication framework based on some kind of Message-Oriented Middleware (MOM), something fully distributed IMs usually expose as a built-in feature, as MOMs typically are a core building block of such solutions.

A Cloud-oriented one fits the scenario, meeting the aforementioned requirements by default in order to cater to the originally intended user base, while at the same time also addressing other more subtle functionalities, such as a tenant-based authorization framework, where several actors (owners, administrator, users) and their interactions with infrastructure may be fully decoupled from the workflows involved (e.g., transfer, rental, delegation). Bonus points include recycling existing (compute/storage-oriented) deployments, getting most visualization and monitoring technologies for free, as those are typically already available in such systems, possibly even enabling federation of different administrative Cloud-enabled domains.

In this sense, our choice leans towards OpenStack [26], as a centerpiece of infrastructure Cloud solutions for most commercial, in-house and hybrid deployments, as well as a fully OpenSource ecosystem of tools and frameworks upon which many EU projects, such as CloudWave (FP-7) [27], are founding their Cloud strategies. Moreover, with an independent consortium featuring a diverse partnership, including world-class industrial players and smaller enterprises, and a global community of thousands developers, OpenStack has the clout to withstand most shifts in paradigms and approaches, and holds the promise to keep its relevance in time within the increasingly crowded Infrastructure as a Service space.

As a reference with respect to the IoT nodes to be connected to the service-oriented Cloud infrastructure, we take into account the latest *Arduino YUN* [28]—like boards. Such kind of devices is usually equipped with (low power) micro-controller (MCU) and micro-processor (MPU) units. Typically, they can interact with the physical world by means of a set of sensing and actuation resources connected to their digital/analog I/O pins. Moreover, they can connect to the Internet thanks to Ethernet and WiFi network interfaces. Such kind of smart boards marry the ease of programming the MCU (typically with simple languages derived from C/C++) with the power of a Linux distribution running onboard on the MPU. This allows not only to sense the physical world and actuate on it, but also to partially elaborate information on-site taking decisions and resorting to external communication only if necessary. In our case, the Linux environment is essential in order to host a complex, plugin-based system, and provide several runtimes for high-level scripting languages.

### 4.2. Data Collection and Inference

**Figure 4:**
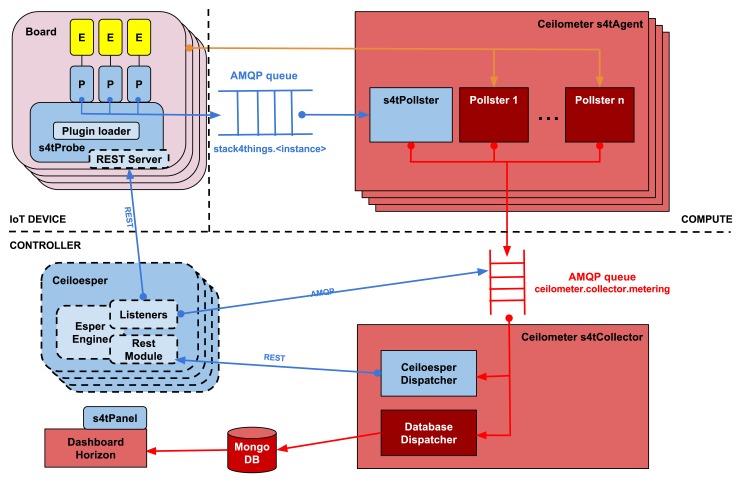
Data collection and inference/reaction subsystem: architecture.

As depicted in Figure 4, the whole Stack4Things data collection architecture comprises both additional modules for Ceilometer and external components needed for higher-level functions, e.g., event processing. Framing the discussion in terms of the devices involved and of compute-based IaaS, the board may be considered an instance of a machine, e.g., a VM or even just a cloud-provisioned physical machine. Along the same lines, a Compute node is just any machine, which, on the one hand, may host standard computing VMs and, on the other, may be IoT-enabled in order to supervise and track the lifecycle of one or more boards. The Controller, in turn, is expected to host a Collector (for the Ceilometer), as a centralized component for data collection, storage or further processing, if needed.

As discussed earlier, the main requirement that we took into consideration while designing the architecture reported in Figure 4 is scalability. In fact, scalability is an essential challenge of smart city-wide experimentations, and it is achieved by allowing one to spin out as many Ceilometer s4t Agents (and CEPs) as needed according to the number of boards actually registered to the system. In huge and complex scenarios, replication of the Ceilometer s4t Collector and MongoDB can be exploited to deal with the amount of data to be collected.

#### 4.2.1. Board-Side: s4tProbe

A probe (s4tProbe), to be hosted in an active instance of a runtime, has been implemented in Python, based on the stevedore library for enabling and loading a monitoring plugin. There is such a plugin for each runtime environment (depicted as *E*), such as Node.js or Python, available on the sensor board, in our case an MPU-equipped Linux-hosting Arduino embedded system, the Arduino YUN model. The same tools and approach are employed to load other OpenStack modules, such as the *pollster*, the *dispatcher, etc.*, in particular with regard to the Ceilometer, in turn made up of an *agent* and a *controller*. More in detail, an abstract class (*PluginBase*) has been designed for the implementation of new plugins and is to be enabled by the aforementioned library for plugins.

To enable communication toward the Ceilometer Agent for the Compute node, the queue-based Apache Qpid messaging system has been employed, providing an implementation of AMQP (Advanced Message Queuing Protocol), an open standard application layer protocol for message-oriented middleware, a bus featuring message orientation, queueing, routing (including point-to-point and publish-subscribe), reliability and security. In particular, the circle-edged arrows represent the interaction model, where there are bus-enabled queues, one or more publishers, as well as one or more subscribers, respectively depicted as pointing toward the queue and out of the queue. For each instance, a message queue gets initialized automatically (if not already available), featuring a name space for addressing under the AMQP environment according to the following format:

stack4things.<INSTANCE>

A RESTful approach, layered over CoAP, has been followed instead for exposing the actuators as corresponding endpoints, whose interactions are represented by arrows pointing to RESTful interfaces. As soon as the Esper Engine detects an event for which an action has been set to be triggered, Ceiloesper sends the corresponding command as a request to one of the aforementioned REST endpoints.

#### 4.2.2. Compute Node-Side : s4tAgent

On OpenStack Compute nodes, a Qpid server had to be installed as a prerequisite with the corresponding Python modules, usually already available by default on the Controller, as there Qpid is needed for the communication between Ceilometer Agent and Collector.

In order not to twist the OpenStack architecture, we opted to implement a *pollster*, e.g., a polling-based sample dequeuing object, which is one of the officially sanctioned methods typically suggested to extend Ceilometer for the monitoring of extra metrics, *i.e*., not measurable through LibVirt, such as the ones exposed by default. This component (/usr/lib/python2.6/site-packages/ceilometer/compute/ pollsters/s4tpoll.py) is to be executed after a fixed amount of time by OpenStack, according to the configuration (/etc/ceilometer/pipeline.yaml) of “pipelines”. A connection would be established to the AMQP queue of each Stack4Things instance running on that Compute node inside this new *pollster* by using Qpid methods. *The pollster* would then go on to extract from queues the messages bearing the samples sent by Probe plugins. The information of interest thus obtained would be used to build a Ceilometer-compliant “sample”, by means of the function util.make_sample_from_instance.

Each sample would then be processed by the Ceilometer Agent to be sent in turn to the Ceilometer Collector hosted on the Controller, by means of the *ad hoc* queue ceilometer.collector.metering. To enable the new *pollster* at boot time for the Ceilometer Agent, the following line has to be added into section [ceilometer.poll.compute]:

s4tpoll=ceilometer.compute.pollsters.s4tpoll:S4tPollster

inside the corresponding file (/usr/lib/python2.6/site-packages/ceilometer-2013.2.3-py2.6.egg-info/ entry_points.txt).

#### 4.2.3. Controller-Side: s4tCollector

To send Stack4Things measurements, as received from the Collector to an inference engine for the infrastructure, called Ceiloesper (developed during the CloudWave project), a new *dispatcher* (CeiloesperDispatcher) for the Collector had to be implemented.

The latter features by default two kinds of *dispatchers* for writing Ceilometer measurements either on file (not depicted in figure, as it usually disabled by default) or in a DB (MongoDB by default), respectively. Even in this case, the addition of our new (CEP-oriented) *dispatcher* did not lead to any modification to the core setup of Ceilometer or OpenStack overall. This new *dispatcher* would analyse only measurements coming from probes and encoded as proper samples by the Ceilometer Agent. More in detail, upon creation of the sample at the Ceilometer Agent, the field meter_dest:ceiloesper would be added into section resource_metadata of the corresponding JSON. Such a field would aid in distinguishing Ceilometer samples from those generated through Stack4Things additions and meant to be consumed by Ceiloesper.

The forwarding of measurements to Ceiloesper would work over REST by sending a JSON message. Ceiloesper is a service built upon Esper, a Java-based CEP (Complex Event Processor) for the detection and management of complex events. This is built by implementing an Esper listener for each event of interest. Event triggering may be based on thresholds or pattern matching for events of higher complexity; such rules are described in the Event Processing Language (EPL), similar in form to SQL queries. At the moment, two listeners related to streams of homogeneous measurements (in our case, either ambient light or noise levels) have been implemented already:

- detecting levels in excess of a certain threshold;
- detecting levels obtained again below the threshold, by pattern matching.

Ceiloesper implements a built-in REST module (a server) listening for measurements sent by the *dispatcher* we wrote for the Collector (CeiloesperDispatcher). Upon arrival of each measurement, an “event object” (called a bean), relevant to the metric to be monitored, would be instantiated, whose fields are to be populated with values extracted from the incoming JSON message. The relevant listener of such an object class would detect the construction of the new object and check whether the EPL rule has been met or else. Indeed, if the rule is respected, an event trigger would fire, and a signal, encoded as an AMQP message and enqueued in the *-metering topic on the Controller, and to be consumed by the Collector, would be sent to the Ceilometer Collector, which would manage it as a Ceilometer sample and write it into MongoDB, as such, from this point onwards, available to be rendered in the dashboard, as any other kind of measurement.

### 4.3. Complex Event Processing

Our modifications to the Esper engine include a built-in REST client to trigger a reaction according to an event of interest and a “container” class for objects (beans) associated with categories of events. The main package is called IoTep, and each bean hosts the attributes that describe the category of objects upon which one or more events are to be processed. Beans have to also host “getter” methods for each attribute to be referenced in an EPL-encoded rule. In particular, Ceilosample is the bean that describes an object defined as a metric received from the CEP with generic attributes that any kind of measurement needs to include (at least: name, metric unit, value, timestamp) and the corresponding getter methods (getName, getUnit, getValue, and so on). A separate package hosts listeners, where each file contains the class that describes an individual event and its handling. Inside such classes, the following methods are to be implemented:

- getStatement(): where the EPL rule corresponding to the event is described.
- update(): where the logic for reaction, to be triggered at the occurrence of the event, described in the getStatement method, is implemented.
- other user-defined methods related to the event.

### 4.4. Visualization: Dashboard

Apart from web UI-based administration tasks, the OpenStack Dashboard, codenamed Horizon, is employed also for data visualization duties. In order to add a real-time graph and a Google Maps-powered sensors geolocation system to Horizon, an IoT-enabled panel, the s4tPanel, has been implemented, included inside the “admin” project label group and labelled “IoT”. The latter requires dropping in a predefined directory (/usr/share/openstack-dashboard/openstack_dashboard/ dashboards/admin) in a folder (iot) that contains all of the essential files, such as:

- panel.py to set the name of the panel and relative access permissions;
- tabs.py to create one or more tabs inside the panel where all graphs are located (in our case, only a “Stats” tab has been created);
- tempiates/iot/stats.html to add JavaScript code related to real-time graphs and Google Maps diagrams, but also to specify the position of the relative “div” sections inside the HTML page.

The dashboard.py file, available in the admin folder described before, needs to be modified, in order to enable the Dashboard to show panels on the left sidebar. Other files involved in this scenario are the ones used to retrieve information from the database by sending a request through the cURL tool and then to parse the response to return data to the JavaScript code in order to plot graphs:

- retrieve.sh to get the admin token, which will be used to retrieve metrics of the corresponding sensors and actuators (ambient light, noise level, as well as number of resources and their Google Maps coordinates);
- chart.php and gmap.php to respectively parse data related to real-time and mapping metrics.

## 5. The System at Work

The Stack4Things architecture has been designed to be applied also to the sensing and actuation scenario involving lampposts described in Section 3.

**Figure 5:**
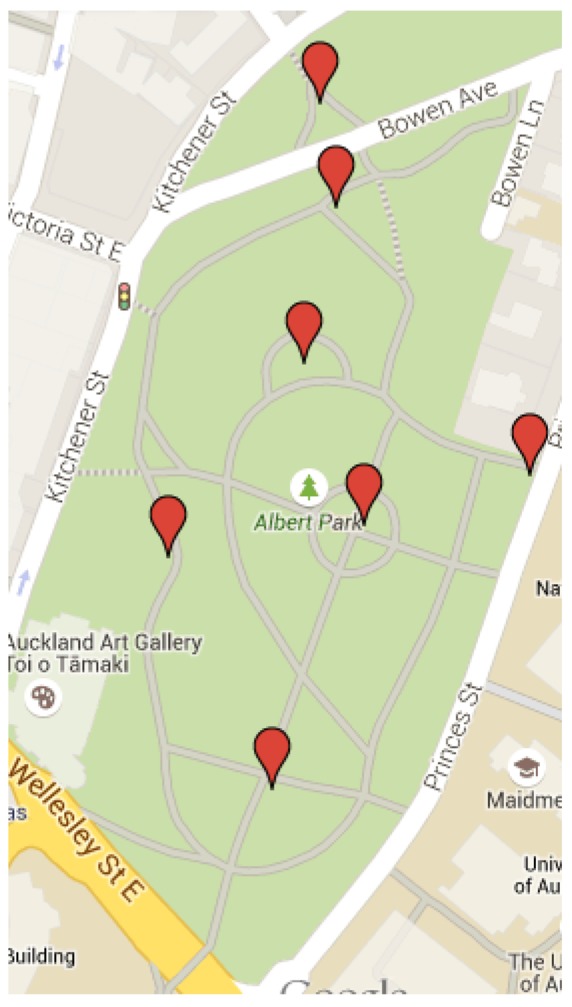
Maps screenshot: smart lampposts locations.

In the screenshot of Figure 5, a map of a section of the park with red markers is depicted, highlighting the locations of select lampposts, the ones that have been enabled for the first iteration of the smart deployment. We used the Google Maps API to geolocate sensors and actuators, whose coordinates are sent inside the resource_metadata JSON field inside Ceilometer metrics.

### 5.1. Design Guidelines

As for required devices, apart from the choice of an embedded system platform for smart sensing, such as Linino, thus demanding boards like the YUN, the following devices may be employed as sensors and actuators to be attached to the cloud-enabled board installed onto the lamppost:

- LDR APDS-9007 as the light sensor;
- LM386 (electret microphone) as the sound sensor;
- MR 16 LED as the lighting element.

Application-specific requirements relative to the design of the system include:

- Setting and operation controls for sensors and actuators, according to resource-specific parameters and specifications:
  – Lamp: allowance for dimming.
  – Acoustic sensor: sound intensity indicated in dBA.
  – Light sensor: voltage-to-lux curve.
- Clamping on data growth, by limiting capture activities and stream uploads to selected time intervals triggered by special conditions, such as presence detection, e.g., associated with significant sound intensity.
- Activation of the full-throttle intensity of lamps as a predefined reaction to potential danger events, when ambient light sensing is indicating that the area is relatively dark.
- Energy saving for the lighting subsystem, by setting illumination levels according to ambient light conditions.

### 5.2. Real-Time Sensing Data and Events

In our tests, we collected measurements related to brightness and noise levels from two peripheral sensors connected to each smart lamppost node. Periodically, with a user-selectable frequency, a request to the MongoDB is sent to retrieve information on the last sample available for each of the aforementioned measurements.

**Figure 6:**
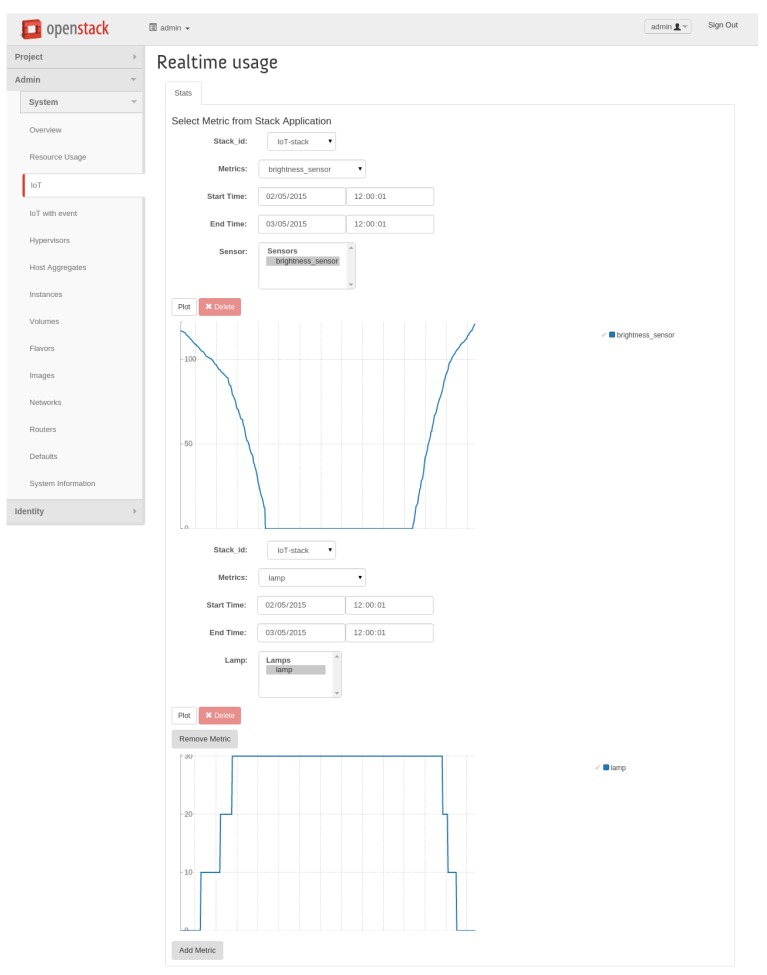
Horizon-IoT panel: real-time graphs (no events).

In Figure 6, there is a screenshot that shows the overall panel layout and how metrics (and events, when selected) are plotted in real time. As long as there are new incoming values to be plotted, lines are updated accordingly, and event-centred bars are drawn onto the event graph, respectively. In this case, the upper line represents the environmental brightness, starting from midday and drawing the next 24 h of samples, whereas the lower one draws the levels at which the lamp is set to operate under normal conditions, as derived from environmental brightness sampled values according to a predefined logic, as explained in Section 5.3.

**Figure 7:**
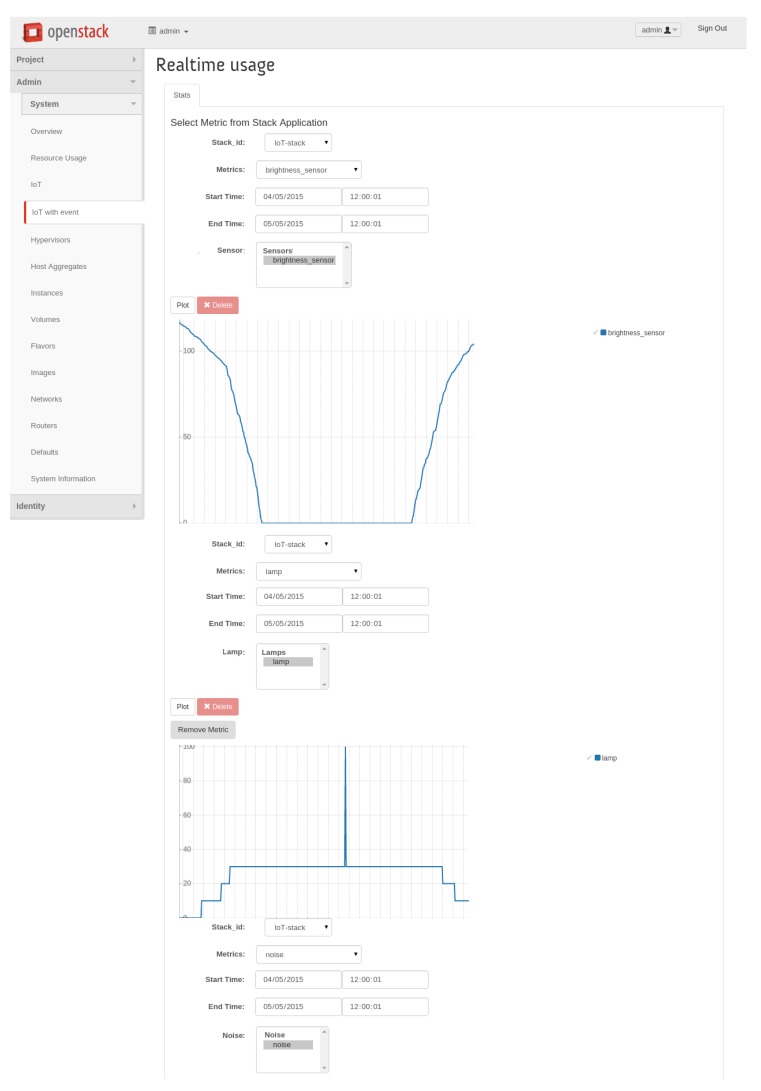
Horizon-IoT panel: lighting real-time graphs (including events).

In Figure 7 instead are graphed the same kinds of metrics, but this time in the presence of an event, due to a distinct loud noise (e.g., someone screaming), which is depicted as a spike in terms of lamp brightness level.

**Figure 8:**
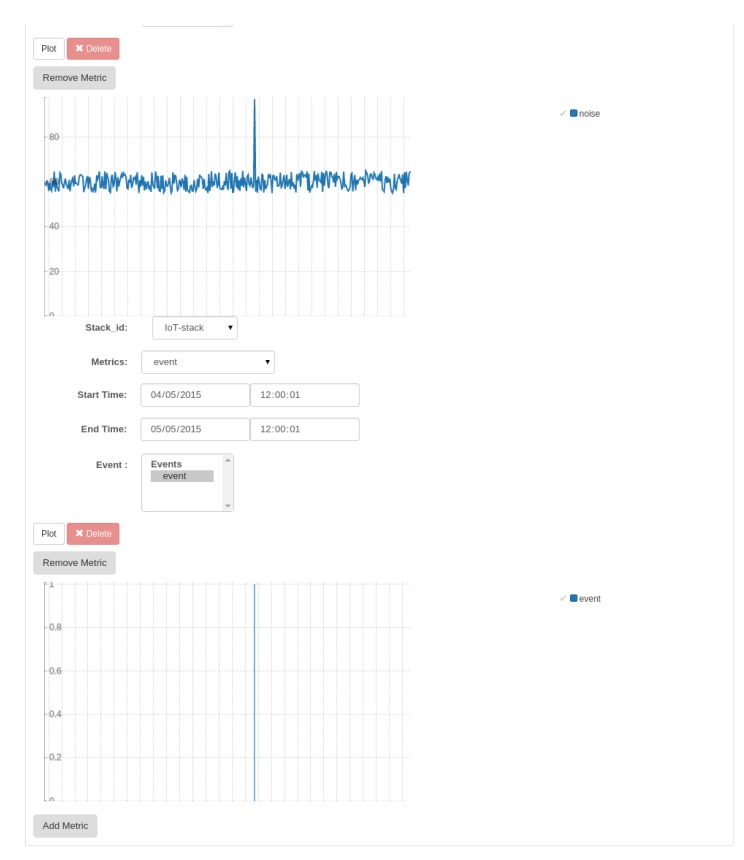
Horizon-IoT panel: noise/events real-time graphs (second half of the page).

Indeed the whole picture is not complete without scrolling down the page in order to catch the other two graphs, as can be seen in Figure 8. The upper one is about the environmental noise levels, typically background noise if we exclude the approximate time of the event, where obviously a spike in the level is recognizable. The lower one is just a depiction of events, as bars centred around the time when such events are detected.

### 5.3. CEP Logic: Statements

As soon as the complex event processing engine is configured and running, we just need to upload the right statements that encode the detection and/or reaction logic. In particular, Code 1 contains the EPL rule triggering the detection of an event (light intensity below a minimum set value or above a corresponding maximum one) only after five (consecutive) matching samples, in order to avoid detecting spurious events. This rule is needed in order to save energy and only switch on the lamp (or increase the brightness stepwise) at twilight and night-time.

**Code 1.** Source code snippet of an EPL rule for light intensity change detection.


String ceilometerEventExpression =“SELECT * FROM event.Ceilosample(sample_name='light_intensity').win:length_batch(5) ”+ “match_recognize ( ”+ “  measures A as luml, B as lum2, C as lum3, D as lum4, E as lum5”+ “  pattern (A B C D E) ”+ “  define ”+ “   A as A.measure <= “ + light_intensity_Max + ”, ”+ “   B as B.measure >= “ + light_intensity_Min + ” AND B.measure <= “ + light_intensity_Max + ”, ”+ “   C as C.measure >= “ + light_intensity_Min + ” AND C.measure <= “ + lightintensity_Max + ”, ”+ “   D as D.measure >= “ + light_intensity_Min + ” AND D.measure <= “ + lightintensity_Max + ”, ”+ “   E as E.measure >= “ + light_intensity_Min + ” AND E.measure <= “ + lightintensity_Max + ”) ” ;

In Code 2 is the EPL rule, which just triggers an event when the noise level goes above a given threshold, in order to detect, e.g., a potential danger.

Code 3 describes the code needed to send a REST request for setting the lamp at maximum brightness when an event is occurring and to keep it at that level for 5 min before restoring a standard level corresponding to the environmental values being sampled, per Code 4.

**Code 2.** Source code snippet of an EPL rule for the detection of a noise level over a certain threshold.


String ceilometerEventExpression=“SELECT * FROM event.Ceilosample(sample_name='noise_level').win:length_batch(1) ”+ “WHERE measure >= noise_threshold”;

**Code 3.** Source code snippet of the actuation logic: lifting the lamp brightness up when a noise-related event is detected.


//set lamp brightness to the maximum valueReguest(“http://212.189.207.109:8888/command/?board=boardnamel&command=analog&pin=Pll&val=100”);//wait 5 mintry { Thread.sleep (5*60*1000);}catch (InterruptedException ex) { Logger.getLogger(NoiseListener.class.getName() ) .log(Level.SEVERE, null, ex) ;}//set lamp brightness to the correct value accordingly to the timeLuxListener.RestoreLightintensity ();

**Code 4.** Source code snippet for functions restoring the lamp brightness to the correct value according to the time.


public static void RestoreLightintensity () { ComputeLightintensity((light_intensity_Min + light_intensity_Max) / 2);}public static void ComputeLightintensity(int lum) { final int minLum = 100; final int maxLum = 400; final int minLumNight = 30; final float divisor = (maxLum - minLum) / 100; float light = (maxLum - lum) / divisor; SimpleDateFormat ft = new SimpleDateFormat (“HH”); Date dNow = new Date (); int daytime = Integer.parselnt(ft.format(dNow)); //compute the correct lamp brightness accordingly to the time if (daytime >= 20 ‖ daytime < 6) {  if (daytime >= 2 & daytime < 6)   light = 30;  else if (light < minLumNight)   light = minLumNight; } else if (lum <= minLum)  light = 100; else if (lum > maxLum)  light = 0; // Set lamp brightness to light percentage Reguest(“http://212.189.207.109:8888/command/?board=boardnamel&command=analog&pin=Pll&val=“; + light);}

## 6. Conclusions

The growing attention devoted to smart cities calls for effective ICT solutions able to cope with huge quantities of devices that need to be managed to provide smart services to citizens. In this paper, we proposed to adopt a new, device-centric approach, allowing one to control geographically-distributed devices, sensing resources and things from the application. The idea is to revert the current approaches mainly focused on data gathering, management and processing, *i.e.* data-centric, providing customization facilities able to also address actuation issues, as well as to inject customized code into sensing resources, thus closing the loop in smart city scenarios. We implemented this approach in Stack4Things: the first attempt at using OpenStack as the underlying technology for the management of IoT devices according to a cloud-oriented approach. We described the architecture of the system by focusing on data management and visualization aspects, while also presenting some implementation details. A running example involving a smart lighting system for citizen safety within the premises of city parks has been presented.
